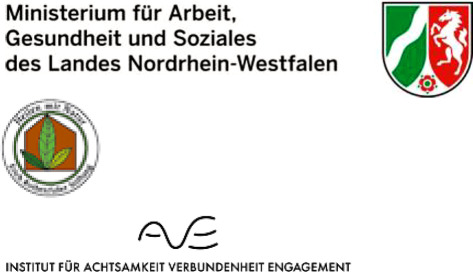# *„Was braucht ihr, damit ihr gern zur Schule kommt?“* Achtsamkeitsbasierte Bildung in Schulen fördert Gesundheit, partizipative Beziehungs- und Kulturgestaltung sowie Demokratiefähigkeit

**DOI:** 10.1007/s11612-022-00648-3

**Published:** 2022-11-28

**Authors:** Nils Altner, Bettina Adler

**Affiliations:** AG Gesundheit & Prävention, Klinik & Lehrstuhl für Naturheilkunde und Integrative Medizin, KEM | Evang. Kliniken Essen-Mitte/Universität Duisburg-Essen, Am Deimelsberg 34a, 45276 Essen, Deutschland

*„Es war die Barbarei, gegen die alle Erziehung geht. Man spricht vom drohenden Rückfall in die Barbarei.“*

Adorno (2012, [1966], S. 125)

## Kontext: Gesunde PädagogInnen brauchen und schaffen gesunde Schulen

In der Klinik für Naturheilkunde und Integrative Medizin der Evang. Kliniken Essen-Mitte werden Menschen mit chronischen Erkrankungen seit 1999 dazu befähigt, die Gesundheitsressourcen ihres Organismus im Alltag zur Wirkung zu bringen. Das Erleben und Erlernen von alltagstauglicher, menschengemäßer und naturnaher Ernährung, Bewegung und Spannungsregulation gehören zum Lehrplan in eigener Sache genauso wie die bewusste Gestaltung von Beziehungen, Werten und Lebensentwürfen. Dabei spielt die Kultivierung von selbstmitfühlender Präsenz eine zentrale Rolle. D. h. die PatientInnen lernen, wie sie im Alltag ihre Aufmerksamkeit immer wieder auf die gegenwärtigen Sinneseindrücke lenken können, um damit für ein paar Atemzüge oder länger freundlich und empathisch zu verweilen.

Zu den PatientInnen der Klinik zählen Menschen, die in sozialen und pädagogischen Berufen arbeiten. Häufig leiden sie darunter, dass sie in ihrem Berufsalltag mehr Kraft und Lebendigkeit verausgaben, als sie regenerieren können. Zu den Belastungen, die immer wieder genannt werden, gehören als abwertend erlebte kollegiale Beziehungen und fehlende Mitgestaltungsmöglichkeiten. Dieses Defizit zwischen Verausgabung und Regeneration trägt über Jahre und Jahrzehnte dann zur Ausprägung von Erkrankungen wie Migräne, Bluthochdruck, chronisch entzündlichen Zuständen, Schmerzerkrankungen oder auch Burnout und Depressionen bei. Gegen Ende des Klinikaufenthalts äußern PatientInnen immer wieder ihr Bedauern und Unverständnis darüber, dass die heilsamen Selbstfürsorgestrategien, die sie sich während ihres Klinikaufenthalts in wenigen Wochen angeeignet haben, ihnen nicht bereits viel früher in ihrem Leben zugänglich waren. Diese Erfahrung haben wir zum Anlass genommen, unsere Aktivitäten in präventive und ressourcenfördernde Schulprojekte zum Wohle der Pädagoginnen und Pädagogen, aber natürlich auch der Schülerinnen und Schüler einzubringen. So haben wir in dem vom Land Nordrhein-Westfalen geförderten Projekt „GIK-Gesundheit, Integration und Konzentration“ mit allen Grundschulen der Stadt Solingen in den Jahren 2016–2018 entsprechende Weiterbildungsformate entwickelt. Die Erfahrungen dort zeigen, dass Lehrerinnen und Lehrer, die lernen, die Beziehung zu sich selbst und ihre Alltagsgestaltung fürsorglich und gesundheitsfördernd zu gestalten, diese Qualitäten dann auch im Miteinander, in den Beziehungen zu den Kindern und in der Gestaltung ihrer Organisationskultur umsetzen (Altner et al. 2018).

## Die aktuellen Bildungsangebote

Im Anschluss an das GIK Projekt wurde von der AG Prävention und globale Gesundheit an den Klinken Essen-Mitte/Universität Duisburg-Essen ein Schulungsprogramm für weitere interessierte Lehrerinnen und Lehrer entwickelt. Diese ca. 50-stündige Weiterbildung „GAMMA-Gesundheit, Achtsamkeit und Mitgefühl im menschenbezogenen Arbeiten“ verbindet Elemente des Stressbewältigungsprogramms MBSR (mindfulness-based stress reduction) von Jon Kabat-Zinn mit Aspekten der Mitgefühlsschulung sowie der Organisations- und Kulturentwicklung nach Otto Scharmer (2019). GAMMA wird als anerkannte schulische Präventionsmaßnahme auf der Grünen Liste des Landespräventionsrates Niedersachsen in Kooperation mit dem Deutschen Präventionstag geführt (2021) sowie von der Landesvereinigung für Gesundheitsförderung in Thüringen empfohlen (2022). GAMMA zielt auf die (Weiter‑)Entwicklung von Fähigkeiten auf mehreren Ebenen ab. Neben den persönlichen Fähigkeiten der bewussten, empathischen Aufmerksamkeit und Selbstregulation möchte das Programm Verständnis, Verbundensein und Engagement im Kollegium stärken. Und natürlich wird auch geübt, Achtsamkeits- und Mitgefühlsübungen für die Kinder anzuleiten, um die gleichen Qualitäten bei ihnen zu stärken. Ziel ist die Förderung von Gesundheit, (Selbst‑)Verantwortung und Freude am Lehren und Lernen für alle Beteiligten.

Basierend auf den pädagogischen Konzepten von Verkörperung und dem Lernen am Modell geht die GAMMA-Weiterbildung davon aus, dass, wenn die Regulations- und Beziehungsfähigkeiten von PädagogInnen unterstützt werden, dies Gesundheit, Mitgefühl, Wertschätzung, Solidarität, Kooperation und Engagement in und zwischen den Berufsgruppen und damit mittelbar auch bei den Kindern stärkt. Darüber hinaus wurden positive Effekte auf den Erhalt und die Stärkung der gesundheits- und bildungsfördernden Schulkultur erwartet. Zudem bieten sich achtsamkeitsbasierte Verfahren und moderate Bewegung im Kontext der Covid-19 Pandemie für die verstärkte Nutzung im Schulalltag an, da ihre immunfördernden Wirkungen gerade im respiratorischen System nachweisbar sind (Rakel et al. 2013). Neben Fragen der rhythmisierten, diversitäts- und beziehungssensiblen Unterrichtsgestaltung wurden auch Aspekte der Kommunikation und Verantwortung im Kollegium, Fragen der Führungskultur sowie der Entwicklung des Schulprofils thematisiert. Neben Berlin und Essen haben wir GAMMA auch mit Kita-LeiterInnen und ErzieherInnen für den Landschaftsverband Westfalen-Lippe sowie für den Ev. Kirchkreis in Dortmund umgesetzt.

Im Zeitraum von September 2019 bis März 2020 wurden 32 von 50 LehrerInnen und ErzieherInnen der Glassbrenner-Grundschule in einem viermoduligen partizipativen und auf den konkreten Schulalltag bezogenen Präsenzformat und weiteren ca. 90 h Selbststudium und Umsetzungspraxis von TrainerInnen der AG Prävention und globale Gesundheit der KEM/Universität Duisburg-Essen geschult. Gemäß der forschungsethischen und datenschutzrechtlichen Richtlinien für fragebogen- und interviewbasierte Datenerhebungen im schulischen Kontext war die Teilnahme an den evaluativen Befragungen komplett freigestellt und wurde anonymisiert durchgeführt. 23 Personen reichten auswertbare Fragebögen zu beiden Befragungszeitpunkten ein und 20 Personen nahmen an Interviews teil. Parallel zur GAMMA-Fortbildung in Berlin führten wir von August 2019 bis Februar 2020 dasselbe Programm in Essen mit weiteren 24 Teilnehmenden durch. Wir begleiteten es ebenfalls auf freiwilliger Basis mit denselben Fragebögen und luden auch hier nach Abschluss der Fortbildung zu Interviews ein. Der Return lag in dieser Gruppe bei 16 kompletten Datensätzen und acht Interviews. Anders als in Berlin kamen die TeilnehmerInnen in Essen aus unterschiedlichen Schulen und Einrichtungen. Erst mit der Auswertung der Daten einer Stichprobe von insgesamt 39 Personen war eine statistische Power der Daten erreicht, um aussagekräftige Korrelationen der durch die Fragebögen ermittelten Kompetenzveränderungen berechnen zu können. Das Fortbildungskurrikulum sowie die drei HauptreferentInnen entsprachen in Essen dem Kurs in Berlin.

## Die Evaluationsinstrumente

Um die Wirkungen der GAMMA-Fortbildung zu erheben, wurden vor und nach dem Kurszeitraum quantitative Daten mit mittels validierter Fragebögen erhoben und nach Ende des Kurses qualitative Daten in Tiefeninterviews gesammelt. Die Wirksamkeitsstudie verwendete damit ein mixed-methods Design. Mittels online über die Studienplattform UNIpark der Universität Duisburg-Essen verfügbare Fragebögen wurden Daten bzgl. individueller Effekte der Weiterbildung u. a. auf die Ausprägung von Achtsamkeit (KIMS) und Selbstmitgefühl (SCS) erhoben sowie auf Basis des Fragebogens zur Arbeit im Team (FAT) bzgl. der Effekte auf organisationaler Ebene.

Umgesetzt wurde ein Pretest-Posttest-Design mit zwei Messzeitpunkten. Der Erhebung lagen u. a. diese validierten Fragebögen in deutscher Fassung zugrunde:

- Kentucky Inventory of Mindfulness Skills (KIMS) (Ströhle et al. 2010)
- Self-Compassion Scale (SCS-D) (Hupfeld und Ruffieux 2011)
- Fragebogen zur Arbeit im Team (FAT) (Kauffeld 2004)

Diese Instrumente wurden ausgewählt aufgrund der Hypothese, dass die Intervention zu einer Förderung von Achtsamkeit (KIMS) und Selbstmitgefühl (SCS) bei den PädagogInnen führt. Und wenn diese beiden Fähigkeiten der persönlichen Stressbewältigung gestärkt werden, dann, so die nächste Hypothese, gestalten die PädagogInnen ihr berufliches Miteinander im Kollegium und mit den Kindern weniger aus Kampf- und Fluchtreaktionen heraus, sondern mit Wertschätzung und partizipativ co-kreativer Gestaltungsfreude. Daher vermuteten wir positive Effekte auf die wahrgenommene Qualität der Teamarbeit (FAT) sowie Berichte über Erfahrungen von mehr Gemeinsamkeit und einer demokratischeren Organisationsentwicklung.

Qualitative Daten wurden in Form von freiwilligen Interviews mit an der Fortbildung teilnehmenden LehrerInnen und ErzieherInnen nach Abschluss der Intervention erhoben. Dabei wurden die geltenden Richtlinien zum Datenschutz eingehalten. Die qualitativen Interviews fanden in Form von „verkörperten phänomenologischen Dialogen“ statt. Diese von unserer Arbeitsgruppe entwickelte Form der qualitativen Datenerhebung dient der Untersuchung von Phänomenen der inneren Erfahrung und Entwicklung der Befragten (Altner und Adler 2021). Dabei gingen die Interviewer achtsamkeits- und mitgefühlsbasiert vor, indem während des Gesprächs immer wieder empathisch Bezüge zur aktuell verkörperten Erfahrung im Zusammenhang mit den GAMMA-Inhalten thematisierten. Eine befragte Lehrerin teilte z. B. mit, dass sie im Verlauf der GAMMA-Fortbildung ihr Verhältnis zu selbst verändert habe. Um dies genauer zu ergründen, wurde sie von der Interviewerin gebeten, sich ihr Selbst-Verhältnis zu vergegenwärtigen und zu beschreiben, welche Körperempfindungen sie damit verbindet. Die Lehrerin benennt als jahrzehntealtes Muster Nackenschmerzen und Sauersein sich selbst gegenüber, wenn sie eigenen Ansprüchen nicht genügte. Durch empathisches Einfühlen in diesen von ihr beschriebenen alten Zustand und durch phänomenologisch leibbezogene Exploration des noch vorsprachlich unausgedrückten neuen Selbstbezugs kann die fragende Person die befragte bei der Bewusstwerdung noch vorbewusster Erfahrungen unterstützen. Dabei klammert sie eigene Vorerfahrungen und Vorurteile aus. So unterstützt, fand die Lehrerin durch das in Sprache Bringen ihrer alten und neuen Körper- und emotionalen Empfindungen dann zu einem starken Ausdruck für ihr neues Verhältnis zu sich, als sie sagte „*Ich kann mir selbst jetzt eine gute Mutter sein*“. Die Gespräche dauerten circa 30 min und wurden per Telefon von Frau Adler, Herrn Gugel und Dr. Altner geführt. Relevante Passagen der Gespräche wurden transkribiert und aus den einzelnen Beschreibungen Themencluster gebildet. Diese wurden dann inhaltsanalytisch zu übergeordneten Themenaussagen reduziert (Mayring und Brunner 2010).

## Ausgewählte Ergebnisse der quantitativen und qualitativen Datenanalysen

### Die Teilnehmenden

Tab. 1.

**Table 1:** 

Anzahl der Personen in Berlin	Geschlecht	Berufe	Altersspanne	Durchschn. Alter
50 Gesamtkollegium	18 Männer, 31 Frauen, 1 divers	35 PädagogInnen, 15 ErzieherInnen	24–64	46,9
32 Tn an der GAMMA Gruppe	23 weiblich, 9 männlich	19 Pädagoginnen, 4 Lehrer 7 Erzieherinnen 2 Erzieher	33–61	47,1
1 drop out	Weiblich	Lehrerin	–	46
23 Fragebogen-AntworterInnen	15 weiblich, 8 männlich	11 Pädagoginnen, 5 Lehrer 4 Erzieherinnen 2 Erzieher	24–61	48,6
20 Interviewte	13 weiblich, 7 männlich	9 Pädagoginnen, 5 Lehrer 4 Erzieherinnen 2 Erzieher	36–64	48,7
24 Tn der Essener GAMMA Gruppe	21 weiblich 3 männlich	18 Pädagoginnen 2 Lehrer 1 Psychologin 1 Gerontologe 1 Personalleiterin 1 Therapeutin	31–69	48,2
16 Fragebogen-AntworterInnen	15 weiblich 1 männlich	16 PädagogInnen	38–69	51,2
8 Interviewte	7 weiblich, 1 männlich	8 PädagogInnen	34–56	47,5

Die vorliegende Auswertung berücksichtigt aus promotionsrechtlichen Gründen nur die Gespräche mit den Berliner KollegInnen. Die Ergebnisse der acht Interviews mit Teilnehmenden der Essener GAMMA-Gruppe werden in Bettina Adlers Dissertation veröffentlicht.

### Qualitativ ermittelte Kompetenzveränderungen und ihr Zusammenwirken

Für ein Verständnis des Zusammenwirkens der Kompetenzen, die die PädagogInnen durch die GAMMA-Fortbildung v. a. im Schulalltag stärken konnten, führten wir Tiefeninterviews in Form von verkörperten phänomenologischen Dialogen durch. Diesen Reflexionsgesprächen lagen folgende Leitfragen zugrunde:

1. Was dich in der GAMMA-Fortbildung besonders berührt und bewegt?
2. Wechselst du in deinem Alltag bewusst in den Zustand der mitfühlenden Achtsamkeit?
3. Wie bringst du Achtsamkeit und Mitgefühl in deine Arbeit ein?
4. Welche Wünsche hast du?

Die Auswertung der Interviewmitschnitte erfolgte anhand der inhaltsanalytischen Sammlung von Beispielaussagen, deren Zuordnung zu Themenclustern und zu überindividuell verallgemeinerbaren Aussagen über persönliche Entwicklungen, die die PädagogInnen der GAMMA-Fortbildung zugeschrieben haben (vgl. Mayring und Brunner 2010). Die Clusterung und Verallgemeinerung wurde vom Erstautor vorgenommen. Eine Inter-Rater-Reliability wurde durch Rückversicherung mit den beiden anderen Interviewern angestrebt. Alle stimmten nach klärenden Nachfragen der Interpretation zu.

Bei der Auswertung der Gesprächsprotokolle ergab die Zusammenschau der Antworten auf die offene Frage: „Was hat dich besonders berührt und bewegt?“, dass alle interviewten PädagogInnen die in dem halben Jahr gemeinsam erarbeiteten theoretischen Inhalte, die gemeinsam erlebten praktischen Wahrnehmungs- und Regulationsübungen sowie den vertrauensvollen Austausch in der Gruppe als sehr bereichernd erlebten. Sie wurden als alltagstauglich, praxisrelevant und gut übertragbar auf die Arbeit mit den Kindern eingeschätzt. Ganz besonders schätzten die KollegInnen die gewachsene persönliche Nähe und Vertrautheit auch über die professionellen Grenzen zwischen LehrerInnen und ErzieherInnen hinweg. In einigen Interviews wurde auch berichtet, wie seitens der Schulleitung Fragen an die KollegInnen und auch an die Kinder gestellt wurden, was sie brauchen, damit sie gern zur Schule kommen. Ein überraschendes Ergebnis war, dass sowohl aus dem Kollegium als auch von den SchülerInnen Orte und Zeiten für mehr Ruhe gewünscht wurden. Daraufhin erklärten sich zwei Lehrerinnen bereit, regelmäßige Stillephasen in der Aula zu ermöglichen. Lehrerzimmer wurden umstrukturiert, damit sowohl Ruhe als auch Gespräche möglich wurden. Außerdem wurde gemeinsam mit interessierten Kindern ein Ort der Stille auf dem Schulhof geplant und gebaut. Hierfür wurde das Architekturbüro bauereignis.de gewonnen und Förderung vom Berliner Bildungssenat gewährt. An dieser sichtbar und nachhaltig manifestierten Co-Kreation auf ihrem Schulhof beteiligten sich die Kinder mit großer Begeisterung und mit Stolz auf das ästhetisch sehr ansprechende und gelungene Ergebnis. Nach dem Erleben und den Bedürfnissen gefragt, gehört sowie von der Leitung ernst genommen und unterstützt zu werden, diese Erfahrungen geben nach unserer Wahrnehmung Anlass zur Identifikation mit *ihrer* Schule und tragen sowohl für die SchülerInnen als auch für die PädagogInnen sehr dazu bei, gern zur Schule zu kommen.

**Zusammenfassend** lassen sich folgende überindividuell verallgemeinerbare qualitative Aussagen zu den von den GesprächspartnerInnen beschriebenen Kompetenzentwicklungen treffen:

1. Die GAMMA-Fortbildung unterstützt einen ressourcen-fokussierten, selbst-wertschätzenden, freundlichen und verantwortlichen Bezug zum Selbst.
2. Sie stärkt eine persönliche, vertrauensvolle und belastbare Beziehungsgestaltung im Team.
3. Nach der GAMMA-Fortbildung berichten die PädagogInnen darüber, dass sie einfühlender eingehen auf die natürlichen Bedürfnisse der Kinder:
  nach einem rhythmisierten Wechsel von Bewegung & Ruhe,
  nach fokussierter Sinnenwahrnehmung und Verbundensein,
  nach Partizipation und Verantwortungsübernahme.
4. Die interviewten PädagogInnen berichten, dass im Verlauf der GAMMA-Fortbildung durch den Zuwachs an Achtsamkeit und Mitgefühl eine Kultur des unterstützenden Miteinanders in der Schule wächst. Kontinuität und Absprachen sind dabei wichtig.
5. Im Gespräch beschreiben sie, wie die in der GAMMA-Fortbildung eingeübte und gestärkte wertschätzende Selbstreflektion, das persönlich miteinander vertraut Sein und die achtsam mitfühlende Selbstregulation, aber auch der empathische und partizipative Führungsstil des Leitungsteams zu einer Kultur des gemeinsamen und freudvollen Gestaltens des Lebensraumes Schule beitragen.

### Quantitativ ermittelte Veränderungen der ermittelten Werte

Im ersten Schritt der Datenanalyse wurden für alle in der Fragebogenbatterie enthaltenen Konstrukte bezogen auf die Gesamtscores 2‑seitige t‑Tests für abhängige Stichproben durchgeführt, um zu prüfen, ob zwischen dem ersten und zweiten Messzeitpunkt statistisch signifikante Veränderungen beobachtbar sind. Die Tabelle mit den Berechnungsergebnissen verdeutlicht, dass die Ausprägungen der Werte vom ersten zum zweiten Erhebungszeitpunkt signifikant stiegen (Tab. 2).

**Table 2:** 

		Mittelwert	Std.-Abweichung	*N*	Sig. (2- seitig)
*Team-Kultur*	FAT gesamt MZP 2 – FAT gesamt MZP 1	3,89744	10,76983	38	0,030*
*Selbstmitgefühl*	SCS gesamt MZP 2 – SCS gesamt MZP 1	5,58974	14,20289	38	0,019*
*Achtsamkeit*	KIMSgesamtMZP 2 – KIMS gesamt MZP 1	7,43590	15,64709	38	0,005*

*p < 0,05 = statistisch signifikant

Das Ergebnis deutet auf einen signifikanten Zuwachs an Kompetenzen für die Zusammenarbeit im Team, für Selbstmitgefühl und Achtsamkeit hin.

### Zusammenhänge zwischen der Zunahme von Achtsamkeit und Selbstmitgefühl mit der Entwicklung der Zusammenarbeit im Team

Über individuelle Entwicklungen hinaus interessierten uns Erkenntnisse zu Zusammenhängen zwischen den beiden Variablen Selbstmitgefühl (SCS) und Achtsamkeit (KIMS) mit Änderungen von Variablen, die für die Entwicklung der Team- und Organisationskultur relevant sind. Dazu berechneten wir diese Korrelationswerte in den Subskalen des Fragebogens zur Arbeit im Team (FAT) (Tab. 3).

**Table 3:** 

		FAT_ Zielorient Differenz	FAT_ Zushalt Differenz	FAT_ Aufgabe Differenz	FAT_ Verantwtg Differenz	FAT_ gesamt Differenz
*SCS_gesamt* *Differenz*	Korr. nach Pearson	0,300	0,372	−0,271	0,394	0,405
	Signifikanz (2-seitig)	0,063	0,020*	0,095	0,013*	0,010*
	*N*	39	39	39	39	39
*KIMS_gesamt* *Differenz*	Korr. nach Pearson	0,189	0,165	0,070	0,268	0,296
	Signifikanz (2-seitig)	0,249	0,316	0,672	0,099	0,067
	*N*	39	39	39	39	39

*p < 0,05 statistisch signifikant

**Figure 1:**
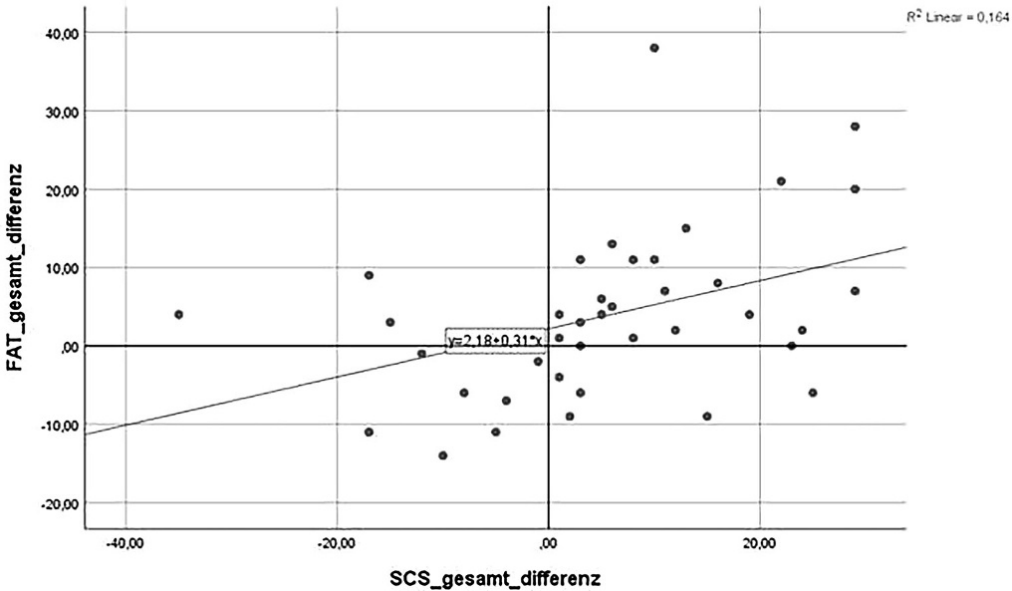

Grafisch ergeben sich folgende statistisch signifikante Einzelkorrelationen (siehe Abb. 1, 2 und 3).

**Figure 2:**
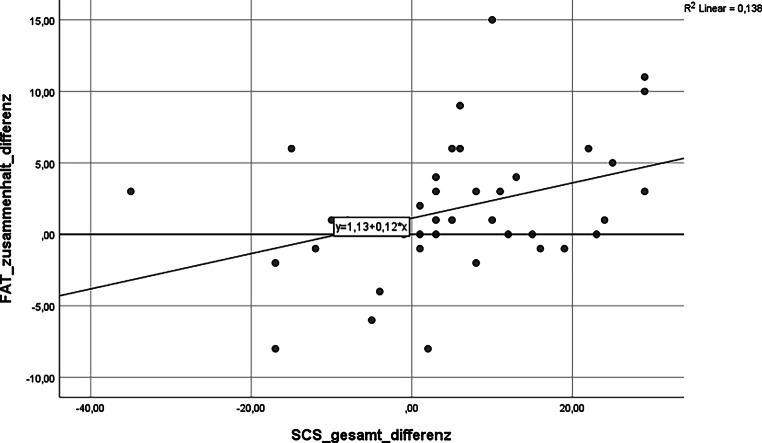

**Figure 3:**
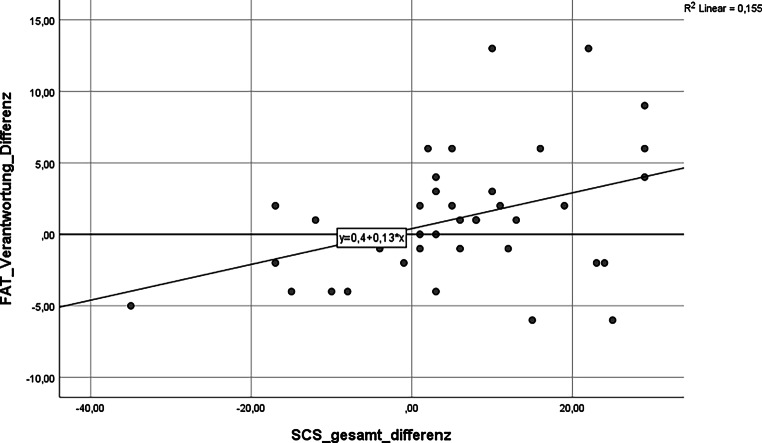

Fazit: Die Entwicklung des Gesamtscores für Teamkultur zeigt eine signifikante Korrelation mit der Zunahme an Selbstmitgefühl (*p* = 0,010). Achtsamkeit und Selbstmitgefühl korrelieren positiv mit allen Subskalen des FAT. **Statistische Signifikanz erreichen dabei die Zusammenhänge zwischen mehr Selbstmitgefühl, steigendem Zusammenhalt im Team sowie der Bereitschaft zur Verantwortungsübernahme im Team.**

Die quantitative Erhebung ergab diese statistisch signifikanten Veränderungen:

- steigende Werte für Achtsamkeit, Selbstmitgefühl, Emotionsregulation, Erholungsfähigkeit und Qualität der Arbeit im Team.
- Mehr Selbstmitgefühl korreliert mit mehr wahrgenommenem kollegialem Zusammenhalt und mehr Bereitschaft zur Verantwortungsübernahme im Team.

## Reflektion: Adornos „Erziehung nach Auschwitz“ 2.0 und die Wendung aufs Subjekt in achtsam mitfühlender Präsenz als Beitrag zu einer friedensfördernden Schulkultur und Organisationsentwicklung

Im Folgenden werden die in der Untersuchung herausgearbeiteten Zusammenhänge zwischen einer gemeinsamen Praxis von Achtsamkeit und (Selbst)Mitgefühl in Schulkollegien und den Auswirkungen auf die Organisationskultur diskutiert. Dabei soll auch die Vision einer friedensfördernden Bildung einbezogen werden, die Theodor Wiesengrund Adorno am 18. April 1966 in seinem Radiovortrag „Erziehung nach Auschwitz“ im Hessischen Rundfunk entworfen hat. Die von ihm identifizierten psychologischen und kulturellen Defizite führten seiner Diagnose nach mit zu den unmenschlichen Greueltaten des zweiten Weltkrieges und der Vernichtungslager. Angesichts des aktuellen Krieges in der Ukraine sowie der antidemokratischen Entwicklungen in Europa und den USA stellt sich uns die Frage, ob die aktuell wieder erstarkenden gesellschaftlichen Tendenzen von Ideologisierung, Radikalisierung, Militarisierung und wachsendem Nationalismus auch heute weiterhin mit den von Adorno identifizierten Phänomenen einhergehen. Er spricht von gesellschaftlichem Druck, dem suppressiven Umgang mit Angst, dem Erziehungsbild der Härte, von Beziehungskälte sowie von einem verdinglichend manipulativen Charakter bei Führungskräften. In unserer Arbeit in Schulen begegnen uns aktuell Hinweise auf genau diese Phänomene. Deshalb möchten wir nachfolgend diskutieren, ob und wie Bildungsangebote zur Kultivierung von Achtsamkeit und Mitgefühl hier transformative Impulse für die Entwicklung von LehrerInnen, SchülerInnen sowie von Schulkultur setzen können und damit gesellschaftlich zur Stärkung humanistischer, friedlicher, demokratiefördernder Haltungen, Verhaltensweisen und Kultur beitragen.

Theodor Adorno (2012 [1966]) benennt in seiner Rede folgende psychologische und gesellschaftliche Erscheinungen, die seiner Meinung nach im ersten Drittel des 20. Jahrhunderts zur Entmenschlichung erschreckend vieler Deutschen beigetragen haben, die dann zu Faschismus, Krieg und Massenvernichtung führten: gesellschaftlicher Druck, die Verdrängung von Angst, das Erziehungsbild der Härte und die Verdinglichung des Bewusstseins. Mitte der 1960 Jahre sieht er sie noch immer präsent (S. 125 f.):

„Der gesellschaftliche Druck lastet weiter trotz aller Unsichtbarkeit der Not heute. Er treibt die Menschen zu dem Unsäglichen, das in Auschwitz nach weltgeschichtlichem Maß kulminierte.“

Erstaunlicherweise erscheint der gesellschaftliche Druck im Jahr 2022 als weiterhin sehr hoch und mit steigender Tendenz. Nach über einem halben Jahrhundert der Entwicklung von Technik, Medizin, Kultur, Politik und Bildung lässt sich weiterhin nicht sagen, dass die Menschen in Deutschland ohne belastenden und oft krankmachenden gesellschaftlichen Druck leben. Das moderne Wort dafür ist „Stress“. Anders als im Jahr 1966 sind inzwischen jedoch individuelle Methoden, mit Stress ressourcenstärkend und gesundheitsfördernd umzugehen, allgemein verfügbar. Unsere Erfahrungen in den Schulen und die Daten unserer Erhebung deuten darauf hin, dass, wenn es gelingt, Methoden wie Achtsamkeits- und (Selbst)Mitgefühlspraktiken gemeinsam zu üben und zu kultivieren, deren Potenzial für die Förderung von Gemeinwohl im Angesicht großer gesellschaftlicher Herausforderungen genutzt werden können. Adorno beschreibt folgende Wirkzusammenhänge, die dafür relevant erscheinen:

„Erziehung müßte Ernst machen mit einem Gedanken, der der Philosophie keineswegs fremd ist: daß man die Angst nicht verdrängen soll. Wenn Angst nicht verdrängt wird, wenn man sich gestattet, real so viel Angst zu haben, wie diese Realität Angst verdient, dann wird gerade dadurch wahrscheinlich doch manches von dem zerstörerischen Effekt der unbewußten und verschobenen Angst verschwinden.“ (Adorno, S. 130 f.)

Die bewusste Hinwendung der Aufmerksamkeit auf innere Gedanken und Gefühle sowie deren Anerkennen und Sein-Lassen zählen zu den Grundqualitäten der Achtsamkeitspraxis. Ihre transformative Kraft liegt genau im akzeptierenden Gewahrwerden der eigenen aktuellen inneren Realität ohne diese verändern oder manipulieren zu wollen. Die Kultivierung von Selbstmitgefühl und Selbstfreundlichkeit im Angesicht der eigenen Ängste bewirkt einen zugewandten, empathischen und fürsorglichen Bezug auch zu den dunklen und problematischen Anteilen der eigenen Persönlichkeit. Nicht das Wegschauen und Verdrängen dieser Anteile trägt zur Entwicklung einer integrierten und souveränen Persönlichkeit bei, sondern die Zuwendung, die Akzeptanz und das bewusste, selbstmitfühlende fürsorgliche damit Sein.

„Dies Erziehungsbild der Härte, an das viele glauben mögen, ohne darüber nachzudenken, ist durch und durch verkehrt (ebd.).“

Die Maxime „Zähne zusammen beißen“, um den äußeren Anschein von Coolness, Unerschütterlichkeit und Leistungsfähigkeit zu wahren, obwohl das Innere Angst, Scham, Trauer, Einsamkeit, Verletztsein oder Verwirrung erlebt, ist uns vor allem in Schulen begegnet, deren Führungsstil wir als autoritär, abwertend und von Konkurrenz bestimmt empfanden. Um dies zu verändern, sind nach unserer Wahrnehmung in erster Linie die Führungskräfte gefragt, ihre eigene Persönlichkeit in einer Weise zu entwickeln, die Ehrlichkeit, Authentizität und ein wertschätzendes, warmes und persönliches Miteinander im Kollegium erlaubt und inspiriert. Zugleich fanden wir bei dem Großteil der Teilnehmenden sowohl in den Gesprächen als auch in der Fragebogenerhebung Hinweise auf die signifikante Zunahme von Selbstmitgefühl. Sich selbst dank der Übungspraxis nun eine gute Mutter oder Freundin sein können, sind Sprachbilder, die uns mehrfach begeistert beschrieben wurden. Die in unseren Daten nachweisbare Korrelation der Zunahme an Selbstmitgefühl mit der Verbesserung der Arbeit im Team zeigt die Auswirkung einer weniger harten, freundlich zugewandten Beziehung zum Selbst auf die Kultur des Miteinander sehr deutlich. In den Schulen, wo wir eine Kälte in den Beziehungen unter den PädagogInnen und auch in ihrem Umgang mit den Kindern spüren konnten, fanden wir dagegen kaum Bereitschaft, miteinander Stille zu erleben, gemeinsam Körperübungen auszuprobieren und über die eigenen Erfahrungen zu sprechen. Adorno beschreibt, warum aber genau solche Anregungen zu Introspektion und vertrauensvollem Austausch sinnvoll sein können:

„Wenn irgend etwas helfen kann gegen Kälte als Bedingung des Unheils, dann die Einsicht in ihre eigenen Bedingungen und der Versuch, vorwegnehmend im individuellen Bereich diesen ihren Bedingungen entgegenzuarbeiten … Das erste wäre darum, der Kälte zum Bewußtsein ihrer selbst zu verhelfen, der Gründe, warum sie wurde.“ (ebd., S. 133 f)

Beziehungskälte in Schulen geht nach unseren Wahrnehmungen mit einem Gefühl der Unsicherheit einher und mit dem Wunsch, möglichst wenig Zeit in der Schule zu verbringen. Besonders für die sehr jungen SchülerInnen, die ein warmes und nährendes Umfeld Zuhause und im Kindergarten gewohnt sind, kann eine solche Atmosphäre traumatisch wirken. Eine Mutter beschreibt das im Gespräch mit uns so:

„Meine Tochter ist im September in die erste Klasse gekommen und das war für sie, die sich darauf total gefreut hat, eine riesige Enttäuschung. Sie hat in den ganzen sieben Jahren ihres Lebens nie so viel geweint wie in den letzten zwei Wochen. Die Lehrerin ist ok, aber leider nicht sehr beziehungsorientiert, und es kommt mir vor, als würde sich mein Kind wie eine Nummer fühlen.“

Gefühlskälte, Technokratie und sich wie eine Nummer zu fühlen, gehört leider noch immer zum Schulalltag vieler Kinder und Erwachsener. Auch Hochschulen und Universitäten sind häufig davon geprägt. Doch sehen Monika Fiegert und Claudia Solzbacher (2014, S. 38 f.) in ihrer historischen Analyse der Entwicklung von Konzepten zu Haltung und Persönlichkeit bei Lehrkräften als Lichtblick, dass in der internationalen Bildungswissenschaft seit dem Jahr 2000 „*nach Jahren eines eher technokratischen Berufsverständnisses … die Bedeutung der Lehrerpersönlichkeit erneut in den Blick*“ kam. „*Unter dem Motto ‚Bildung braucht Beziehung‘ kehrt das Interesse an der Lehrerhaltung … vehement zurück, vor allem beeinflusst durch Erkenntnisse der neurowissenschaftlichen Forschung, die mittlerweile belegen kann, dass die immer wieder betonte wertschätzende, empathische und authentische Haltung der Lehrkraft das Lernen der Schüler fördert*.“

Wenn stattdessen Beziehungskälte und emotionale Härte eine Schulkultur beherrschen, dann geht das nach unseren Erfahrungen meist mit einer starken Betonung von Rolle, Status, Regeln, Konkurrenz und Erfolg einher. Partizipation und Mitgestaltung ist dann kaum möglich und die im Miteinander verwendete Sprache bedient sich dort oft v. a. technisch kühler Begriffe wie z. B. „Mechanismen“, „Hebel“, „Steuerung“, „Programm“, „Maßnahme“, „Messen“, „Effizienz“ oder „Ergebnissicherung“. Sich wie eine Nummer zu fühlen, die stimmen soll und verwaltet wird oder wie ein Rädchen in einem unpersönlichen Getriebe, das zu funktionieren hat, sind Sprachbilder, die das Gefühl des manipuliert Werdens wiedergeben. Adorno (ebd., S. 131) führt in seiner Analyse Forschungsergebnisse zum „manipulativen Charakter“ an, die er und Max Horkheimer in der amerikanischen Emigration erarbeitet hatten:

„Der manipulative Charakter … zeichnet sich aus durch Organisationswut, durch Unfähigkeit, überhaupt unmittelbare menschliche Erfahrungen zu machen, durch eine gewisse Art von Emotionslosigkeit, durch überwertigen Realismus. Er will um jeden Preis angebliche, wenn auch wahnhafte Realpolitik betreiben. Er denkt oder wünscht nicht eine Sekunde lang die Welt anders, als sie ist, besessen vom Willen of doing things, Dinge zu tun, gleichgültig gegen den Inhalt solchen Tuns. Er macht aus der Tätigkeit, der Aktivität, der sogenannten efficiency als solcher einen Kultus, der in der Reklame für den aktiven Menschen anklingt …

Hätte ich diesen Typus des manipulativen Charakters auf eine Formel zu bringen – vielleicht soll man es nicht, aber zur Verständigung mag es doch gut sein –, so würde ich ihn den Typus des verdinglichten Bewußtseins nennen. Erst haben die Menschen, die so geartet sind, sich selber gewissermaßen den Dingen gleichgemacht. Dann machen sie, wenn es ihnen möglich ist, die anderen den Dingen gleich.“

Es ist vor allem die Einladung, gemeinsam im Tun innezuhalten und Stille zu erleben, die in unseren Achtsamkeitsangeboten hier eine im wahrsten Sinne des Wortes „Zumutung“ darstellte. Sie ermöglichte allen, die sich darauf einlassen wollten, sich selbst und einander in einer Zustandsform kennenzulernen, die genau nicht von Manipulation, kaltem Aktionismus und technokratisch verdinglichter Verfügungsgewalt über die eigene Person sowie über die KollegInnen und die Kinder geprägt ist. Statt solch blindem und oft aktionistisch lautem Tun und Machen finden in der stillen Introspektion die unmittelbaren menschlichen Erfahrungen ins Bewusstsein, die sich eher zaghaft, leise und warm im Da-Sein zeigen. Adorno formuliert sein Plädoyer für eine not-wendige Selbstzuwendung so:

„Nötig ist, was ich unter diesem Aspekt einmal die Wendung aufs Subjekt genannt habe.“ (ebd., S. 125)

Immer wieder eine gemeinsame Zeit der Stille und des bewussten Nichtstuns zu erleben, in der die Aufmerksamkeit jeder Person freundlich eingeladen ist, offen, wertschätzend und mitfühlend das wahrzunehmen, was im eigenen Inneren und im gemeinsamen Miteinander aktuell präsent und spürbar ist, kann radikale und mit der Zeit auch kulturbildende „Wurzel“-Erfahrungen ermöglichen. Für den israelischen Bildungswissenschaftler Oren Ergas (2017, S. X) liegt die untrennbare Verbindung zwischen dem „inneren“ Leben und Lernen mit der „äußeren“ pädagogischen Praxis auf der Hand. Er plädiert daher ganz ausdrücklich für eine introspektive Selbstschulung von Lehrenden als Basis für ihre authentisch verkörperte und verantwortliche pädagogische Arbeit:

„This embodied mind … is that which governs the hand that is raised to hit or to caress and the mouth that is open to curse or to bless. You are the only one that has a privileged direct access to your mind as I have to my own. We are the only ones that can take responsibility for it. It seems like we need to engage in a far more rigorous study of the mind and what it brings to the ‚curriculum‘ so that ‚education‘ as a ‚mind-making practice‘ will indeed tackle the problem at its source.“

Wer sich an PädagogInnen erinnert, die im eingenen Leben eine inspirierende Rolle gespielt haben, wird sich in der Regel weniger an ihre Worte erinnern und daran, *was* sie im Detail unterrichtet haben, als daran *wie* wir sie und *wie* wir uns in ihrer Gegenwart erlebt haben. Berühren und inspirieren uns vielleicht besonders die introspektiven, selbstreflektierten PädagogInnen, die ihre Werte und Haltung wirklich verkörpern?

## Fazit und Ausblick: mitfühlende Achtsamkeit und die Bildung von Demokratiefähigkeit

Sowohl im Rahmen des NRW-Landesprojekts „GIK-Gesundheit, Integration, Konzentration“, in den GAMMA-Schulen sowie aktuell mit 21 Schulen im Rheinland im Rahmen des aktuell vom NRW-Ministerium für Arbeit, Gesundheit und Soziales geförderten Projekts „AmSeL-Achtsamkeits- und mitgefühlsbasierte Suchtprävention in Schulen“ in Kooperation mit der update Fachstelle für Suchtprävention in Bonn erleben wir, dass die Praxis von mitfühlender Achtsamkeit in Schulen nicht nur die einzelnen Personen und ihre Beziehungen stärkt, sondern auch kulturgestaltend wirken kann. Voraussetzung dafür ist, dass die Schulleitung einen Führungsstil praktiziert, der Druck und Angst reduziert, der statt Härte und Konkurrenz einen warmen und zugewandt freundlichen, humorvollen, auch herzlichen Umgang im Kollegium pflegt und der statt manipulativ technokratisch zu agieren, ein lebendiges gemeinsam gestaltendes Miteinander ermöglicht und fördert.

Wir sehen hier ein großes Potenzial für die Bildung von Demokratiefähigkeiten. Der NRW-Demokratiebericht (LZpB 2021, S. 35) konstatiert den aktuellen Stand dazu im bevölkerungsreichsten deutschen Bundesland so: „Je näher Demokratie und die damit verbundenen Prozesse (z. B. Konfliktaustrag) rücken und je stärker die eigene Haltung gefragt ist (z. B. Kompromissfähigkeit, Toleranz), desto niedriger ist die Zustimmung“ und fordert: „die lebensweltliche Demokratiebildung (zu) stärken. Die Leitlinie dabei muss sein, dass erst das Erleben von Demokratie sie auch als Lebensform begreifbar macht“. Autokratisch und manipulativ geführte Schulen und Klassen in achtsam, mitfühlend, partizipativ und co-kreativ gestaltete Lern- und Lebensräume zu verwandeln, entspricht ganz diesem Bildungsauftrag.

Doch trotz entsprechender gesetzlicher Vorgaben zur Mitbestimmung, erleben SchülerInnen (und auch LehrerInnen) in Deutschland häufig, dass ihre Mitsprache bei der Gestaltung der Schulkultur unerwünscht ist. *„Dadurch entsteht auch eine Art Demokratieverdrossenheit. Viele glauben, sie können sowieso nichts bewirken, und sind dann offen für rechtsextreme Ideologien“*, sagt eine Schülervertreterin (Anders 2020). Unsere Projektergebnisse legen nahe, dass die gemeinsame mitfühlende Achtsamkeitspraxis dazu beitragen kann, eine Schulkultur zu schaffen, die geprägt ist vom Interesse der Schulleitung und der PädagogInnen an den SchülerInnen und aneinander sowie vom Interesse an den Bedürfnissen und Wünschen aller Beteiligten für eine gesunde, freudvolle und entwicklungsfördernde Schule. In ihrem Artikel über Achtsamkeitspraxis, Demokratie und Bildung erhellen Andrea Marie Hyde und James LaPrad (2015) die Verbindungen zwischen der selbstreflektiven Praxis von Achtsamkeit und dadurch ermöglichten bewusst kulturgestaltenden Haltungen und Handlungen im Bildungskontext. So verstehen sie Achtsamkeit as „a form of critical first-person investigation“ (S. 3), die kritisches Bewusstsein stärkt: „critical awareness requires a practice of continual awakening. Here is where mindfulness, as a pedagogical practice, has a place in democratic education“ (S. 2). Hyde und LaPrad knüpfen dabei an John Deweys Verständnis von Bildung als Befähigung zur kontinuierlichen Persönlichkeitsbildung im Dienste von Gemeinwohl und Demokratie an und an Paolo Freires Pädagogik der Befreiung von Unterdrückung. Weder Dewey noch Freire verwenden dabei den Begriff „Achtsamkeit“, doch beide betonen die Notwendigkeit sowie das persönlich und politisch transformative Potenzial der Bildung von Selbstwahrnehmung und bewusster Persönlichkeitsentwicklung als gelebte Ethik der menschlichen Verbundenheit und Solidarität. Im Kontext des ganz konkreten Schulalltags, so deuten unsere Befunde an, kann die von Adorno benannte pädagogische „Wendung aufs Subjekt“ in Form von Achtsamkeits- und Mitgefühlspraxis LehrerInnen und SchülerInnen dabei unterstützen, to „raise the consciousness of one’s personal and social position by putting one in touch with the world as it really is, through the simple practice of nonjudgemental awareness“, so Hyde und LaPrad (S. 5). Die Haltung des Mitgefühls ist dabei für Hyde und LaPrad „the capacity to feel, and wish to relieve, the suffering of others“ (ebd.).

Damit diese Transformation gelingen kann, braucht es einen langen Atem und das Engagement zumindest einiger PädagogInnen pro Schule für die kontinuierliche und verkörperte Inspiration. Der Schulleitung kommt dabei eine wesentliche Gestaltungsrolle für die Schulkultur zu. Die Bereitschaft und Geschwindigkeit jeder einzelnen Person für achtsames Innehalten, für Innenschau, Öffnung, mitfühlende Begegnung, für vertrauensvolle Gespräche und gemeinsame Gestaltung haben wir in den Schulen dabei als sehr individuell und unterschiedlich erlebt. Freiwilligkeit, Freundlichkeit und Ausdauer sind daher wirklich unverzichtbare Aspekte der transformativen Bildung, die mit der bewusst introspektiven Wendung aufs Subjekt den Boden bereitet für eine humanistische und demokratiefördernde Entwicklung der Organisationen und Kultur. Am Ende unserer Begleitung der Berliner Schule sagte der Schulleiter: „*Dank der GAMMA-Fortbildung haben wir bisher die Corona-Krise mit Gelassenheit und Humor meistern können*“. Das freut uns sehr, wir bedanken uns herzlich für die inspirierende und sinnvolle Zusammenarbeit und wünschen einen weiter guten Weg!